# Nodes of Ranvier and Paranodes in Chronic Acquired Neuropathies

**DOI:** 10.1371/journal.pone.0014533

**Published:** 2011-01-18

**Authors:** Carmen Cifuentes-Diaz, Odile Dubourg, Theano Irinopoulou, Marc Vigny, Sylvie Lachkar, Laurence Decker, Patrick Charnay, Natalia Denisenko, Thierry Maisonobe, Jean-Marc Léger, Karine Viala, Jean-Jacques Hauw, Jean-Antoine Girault

**Affiliations:** 1 Institut National de la Santé et de la Recherche Médicale (Inserm), Unité Mixte de Recherche en Santé (UMR-S) 839, Paris, France; 2 Université Pierre et Marie Curie (UPMC), Paris, France; 3 Institut du Fer à Moulin, Paris, France; 4 Laboratoire de Neuropathologie Raymond-Escourolle, Pitié-Salpêtrière Hospital, Assistance Publique des Hôpitaux de Paris (AP-HP), Paris, France; 5 Consultation de Pathologie Neuromusculaire, Centre de Référence de Paris Est, Pitié-Salpêtrière Hospital, AP-HP, Paris, France; 6 Institut de Biologie de l'Ecole Normale Supérieure (IBENS), Inserm U1024, Centre National de la Recherche Scientifique (CNRS) UMR 8197, Ecole Normale Supérieure, Paris, France; 7 Fédération de Neurophysiologie Clinique and Fédération de Neurologie, Pitié-Salpêtrière Hospital, AP-HP, Paris, France; McMaster University, Canada

## Abstract

Chronic acquired neuropathies of unknown origin are classified as chronic inflammatory demyelinating polyneuropathies (CIDP) and chronic idiopathic axonal polyneuropathies (CIAP). The diagnosis can be very difficult, although it has important therapeutic implications since CIDP can be improved by immunomodulating treatment. The aim of this study was to examine the possible abnormalities of nodal and paranodal regions in these two types of neuropathies. Longitudinal sections of superficial peroneal nerves were obtained from biopsy material from 12 patients with CIDP and 10 patients with CIAP and studied by immunofluorescence and in some cases electron microscopy. Electron microscopy revealed multiple alterations in the nodal and paranodal regions which predominated in Schwann cells in CIDP and in axons in CIAP. In CIDP paranodin/Caspr immunofluorescence was more widespread than in control nerves, extending along the axon in internodes where it appeared intense. Nodal channels Nav and KCNQ2 were less altered but were also detected in the internodes. In CIAP paranodes, paranodin labeling was irregular and/or decreased. To test the consequences of acquired primary Schwann cells alteration on axonal proteins, we used a mouse model based on induced deletion of the transcription factor Krox-20 gene. In the demyelinated sciatic nerves of these mice we observed alterations similar to those found in CIDP by immunofluorescence, and immunoblotting demonstrated increased levels of paranodin. Finally we examined whether the alterations in paranodin immunoreactivity could have a diagnosis value. In a sample of 16 biopsies, the study of paranodin immunofluorescence by blind evaluators led to correct diagnosis in 70±4% of the cases. This study characterizes for the first time the abnormalities of nodes of Ranvier in CIAP and CIDP, and the altered expression and distribution of nodal and paranodal proteins. Marked differences were observed between CIDP and CIAP and the alterations in paranodin immunofluorescence may be an interesting tool for their differential diagnosis.

## Introduction

Chronic polyneuropathy is a highly prevalent condition with various etiologies, including hereditary, metabolic, toxic or immune-mediated origins. For hereditary polyneuropathies, termed Charcot-Marie-Tooth (CMT) diseases, recent advances in the identification of the responsible genes allow a genetic diagnosis in a growing number of cases [1]. Acquired neuropathies are more frequent than hereditary neuropathies and their diagnosis is based on a combination of clinical, electrophysiological, biological and, when necessary, histopathological evidence. Despite thorough investigations, no cause is found in 10–15% of patients with chronic polyneuropathies [2]. Here, we studied two types of chronic acquired polyneuropathies whose diagnosis can be challenging, chronic inflammatory demyelinating polyneuropathy (CIDP) and chronic idiopathic axonal polyneuropathy (CIAP). The distinction is important since specific treatment options are available for CIDP [3], whereas there is none of proven efficacy for CIAP [4].

CIDP is an immune-mediated disorder of peripheral nerves, with a progressive or relapsing course [5], [6]. Its diagnosis is based mainly on clinical and electrophysiological features [5], [7]. In its most common clinical presentation, CIDP combines motor deficits involving proximal and/or distal segments of the limbs, and superficial and/or deep sensory loss, progressing for at least two months [5]. Cerebrospinal fluid (CSF) examination often shows increased protein levels without cells. However, CIDP clinical presentation may be atypical [8], and electrodiagnostic criteria for demyelination may be missing because segmental demyelination affects only a few myelinated fibers or proximal segments not easily studied by electroneuromyography (ENMG). CIAPs are slowly progressive distal polyneuropathies, involving sensory or motor and sensory modalities, for which no cause is found after a thorough biological investigation [9]. In some cases, CIDP may be impossible to differentiate from CIAP on the basis of clinical and electrophysiological examinations, a situation that requires a nerve biopsy [10]. This procedure must be performed by trained physicians and examined in a laboratory with expertise in nerve pathology, as it requires sophisticated approaches, such as electron microscopy of transverse sections of peripheral nerve [10], [11].

Myelinated fibers are characterized by highly differentiated domains along the axon, centered by nodes of Ranvier [12], [13]. The voltage-gated Na^+^ channels (Nav), essential for the rapid saltatory conduction of action potentials, are concentrated at nodes of Ranvier in association with ankyrin G, a cytoplasmic protein interacting with cortical cytoskeleton and cell adhesion molecules [14], and KCNQ2 potassium channels [15]. The paranodal septate-like junctions separate the nodal and juxtaparanodal regions, attach the glial loops to the axonal membrane, and restrict the lateral diffusion of axonal membrane proteins between the node and the internodal space. Several proteins involved in axoglial contacts have been identified [12], [16], including paranodin [17], also known as Caspr [18]. Nodal and paranodal proteins are altered in mouse models of CMT [19], [20], and have been investigated in skin biopsies from CMT patients [21]. However, the possible alterations of nodal and paranodal regions in CIDP and CIAP are not known. The purpose of this study was to investigate the nodal and paranodal regions in these polyneuropathies using both electron microscopy and immunofluorescence in order to better understand their pathophysiology and refine their diagnosis.

## Results

### Nodal and paranodal ultrastructure is differentially altered in Schwann cells and axons in CIDP and CIAP

**Figure 1:**
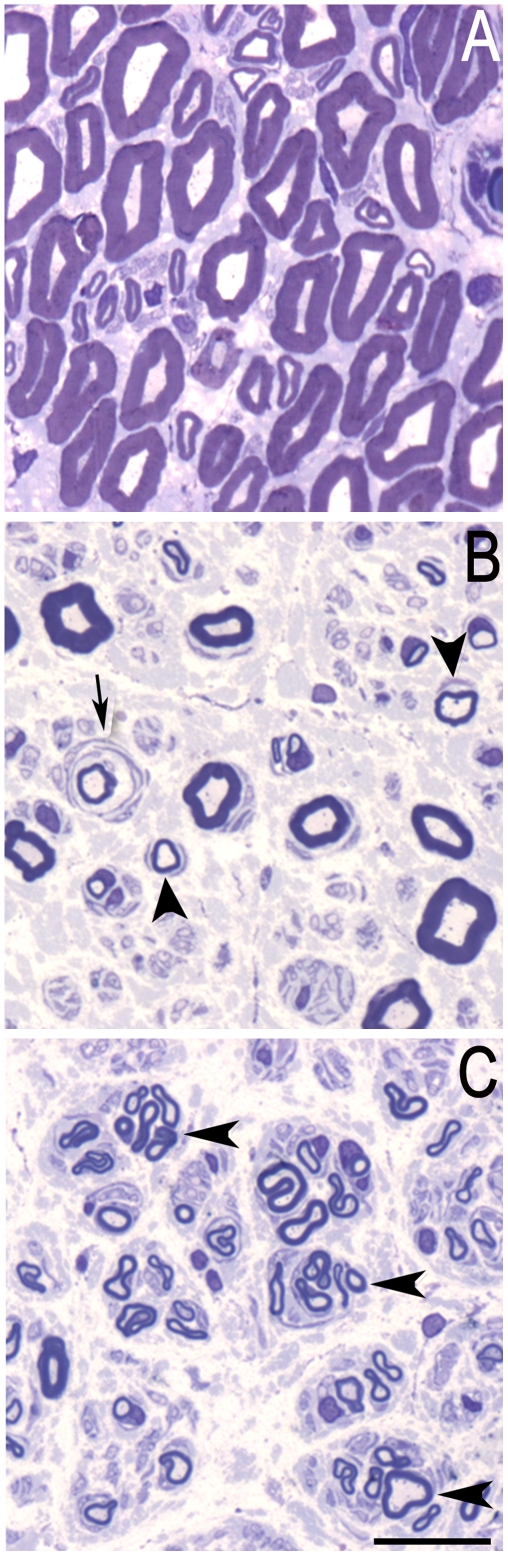
Transverse semi-thin sections of the musculo-cutaneous nerve of a control and patients with chronic acquired neuropathies. **A**. Normal density of myelinated fibers in a nerve without lesion. **B**. In a CIDP nerve (Patient 12), several hypomyelinated fibers (arrowheads) and an onion bulb formation (arrow) are visualized. **C**. In CIAP nerve (Patient 13), loss in myelinated fibers and numerous regenerating clusters (arrowheads) are present. Scale bar 30 µm.

Superficial peroneal nerve biopsies were studied in 12 patients diagnosed with CIDP (Table S1) and 10 with CIAP (Table S2). We first examined toluidine blue staining of transverse semi-thin sections (Fig. 1). In contrast with a sample with no sign of peripheral neuropathy which was considered as a control (Fig. 1A), hypomyelination (Fig. 1B, arrowheads) and onion bulbs (Fig. 1B, arrow) were visible in a typical CIDP sample. The CIAP sample displayed a decreased number of fibers and regeneration clusters (Fig. 1C, arrowheads).

**Figure 2:**
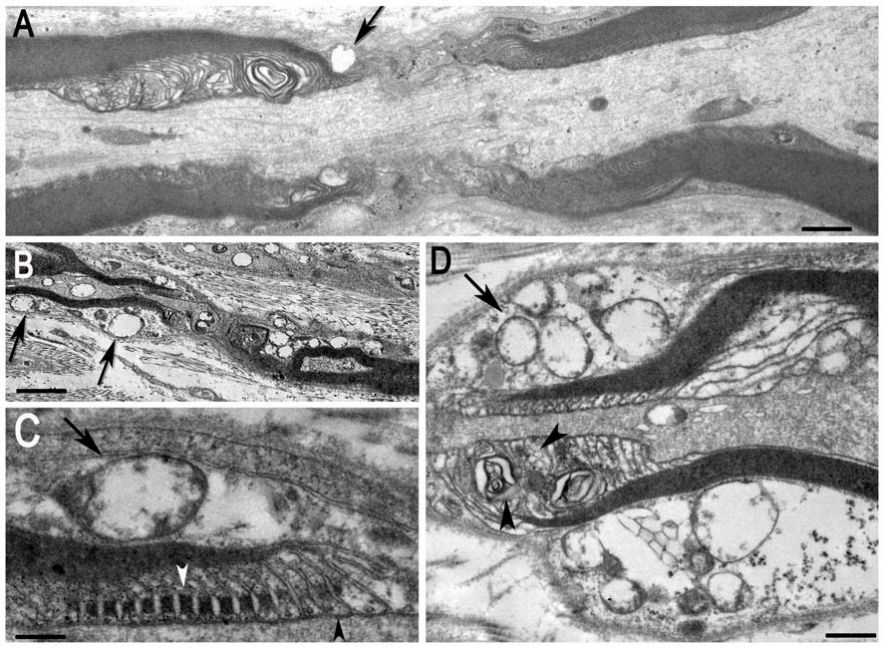
Ultrastructure of nodes of Ranvier in superficial peroneal nerves from 2 CIDP patients. **A**. A myelinated fiber (Patient 6) with a vacuolar inclusion (arrow) in the outer cytoplasm of the Schwann cell. **B**. A myelinated fiber (Patient 9) displaying numerous vacuoles in the cytoplasm of the Schwann cell (black arrows). **C**. Higher magnification showing a vacuole in the Schwann cell (arrow), and intact adherens junctions (white arrowhead) and septate-like paranodal axoglial junctions (black arrowhead) junctions. Note that paranodal loops are regularly spaced and tightly linked to the axolemma (black arrowhead). **D**. Another myelinated fiber from the same patient in which the Schwann cell cytoplasm contains large vacuoles with granular material (arrow), lamellar and multivesicular bodies (arrowheads). Scale bar: A, 0.6 µm; B, 3 µm; C, 300 nm; D, 1 µm.

Since, to our knowledge, the nodal regions have not been explored in CIDP or CIAP, we used electron microscopy to determine how these regions were altered in chronic acquired neuropathies. Ten nodes of Ranvier were examined for either pathology, in longitudinal sections of nerves from two patients with CIDP and two with CIAP. In CIDP myelinated fibers, the abaxonal cytoplasm and the perinodal extensions of Schwann cells were abnormal as well as the paranodal glial loops (Fig. 2). The outer Schwann cell cytoplasm contained multiple large vacuoles sometimes enclosing granular structures (Fig. 2A–D, arrows). The axons were not entirely normal and the nodal axoplasm included some vacuolar structures (Fig. 2B). At the level of paranodes, adherens junctions between adjacent glial loops (Fig. 2C) and axoglial septate junctions (Fig. 2C, arrowheads) were relatively preserved. However, some paranodal loops were of irregular size and some contained lamellar and multivesicular bodies (Fig. 2D, arrowheads).

**Figure 3:**
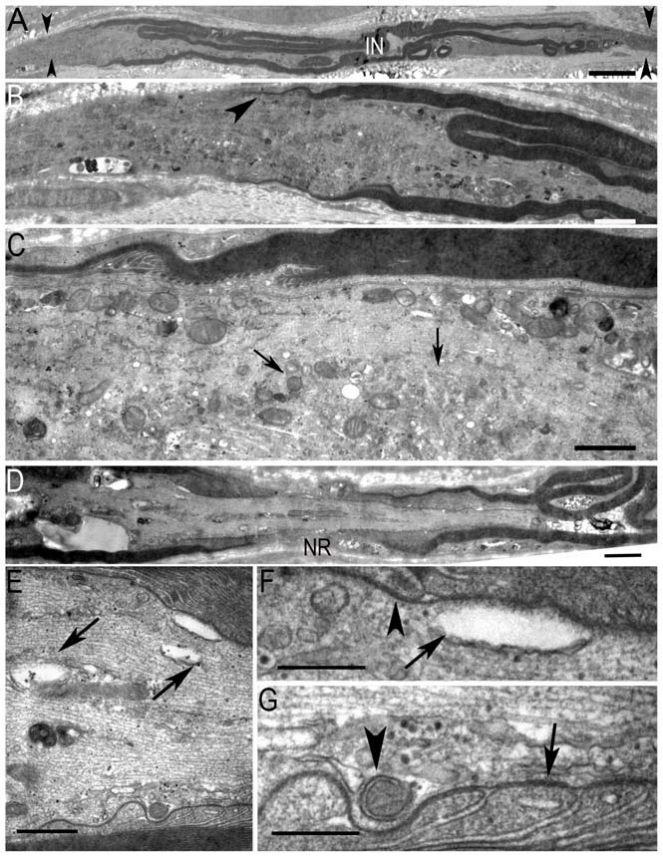
Ultrastructure of nodes of Ranvier in superficial peroneal nerves from 2 CIAP patients. **A**. Low magnification of a fiber (Patient 14) with a short internodal (IN) distance between two consecutive heminodal regions (arrowheads), possibly corresponding to a regenerating fiber. **B**. Higher magnification of the left heminode in A showing the thinly myelinated segment (arrowhead). **C**. Higher magnification of B. Note the presence of numerous organelles in the axoplasm (arrows). **D**. Low magnification of a longitudinal ultrathin section of a node of Ranvier (NR) region (Patient 20). **E**. Higher magnification of D showing axoplasmic vacuoles (arrows). **F**. Higher magnification of E showing paranodal junctions (arrowhead) and a vacuolar space close to the axolemma (arrow). **G**. Another detail of E (bottom right) showing paranodal loops with axoglial junctions (arrow) and axoplasmic protrusions with organelles (arrowhead). Scale bar: A and D: 2.5 µm, B and E: 20 nm, D, E: 10 µm, C, F, and G: 300 nm.

In CIAP nodal regions, the abaxonal glial region and Schwann cell microvilli were normal (Figure S1). Some nodes were heminodes or were flanked by two Schwann cells with different degrees of myelination, some of which had very thin myelin (Fig. 3A–C) suggesting that they corresponded to myelination of regenerating fibers. The axoplasm was rich in organelles (Fig. 3C, arrows). In other fibers vacuoles of various sizes were frequently observed in the axoplasm (Fig. 3D–F). In the paranodal region vacuoles were found in the vicinity of the glial loops, often in regions with poorly defined axoglial junctions (Fig. 3F, arrow), whereas other regions displayed normal septate-like junctions (Fig. 3G arrow, Fig. S1C, arrowhead). The axolemma formed protrusions which contained cellular organelles, mitochondria, and vesicles, some of which possibly corresponded to glial invaginations (Fig. 3G, arrowhead). Thus, electron microscopy showed the existence of multiple abnormalities of nodal and paranodal regions in chronic acquired neuropathies.

### Alterations in paranodin and Nav immunoreactivities differ in CIDP and CIAP

**Figure 4:**
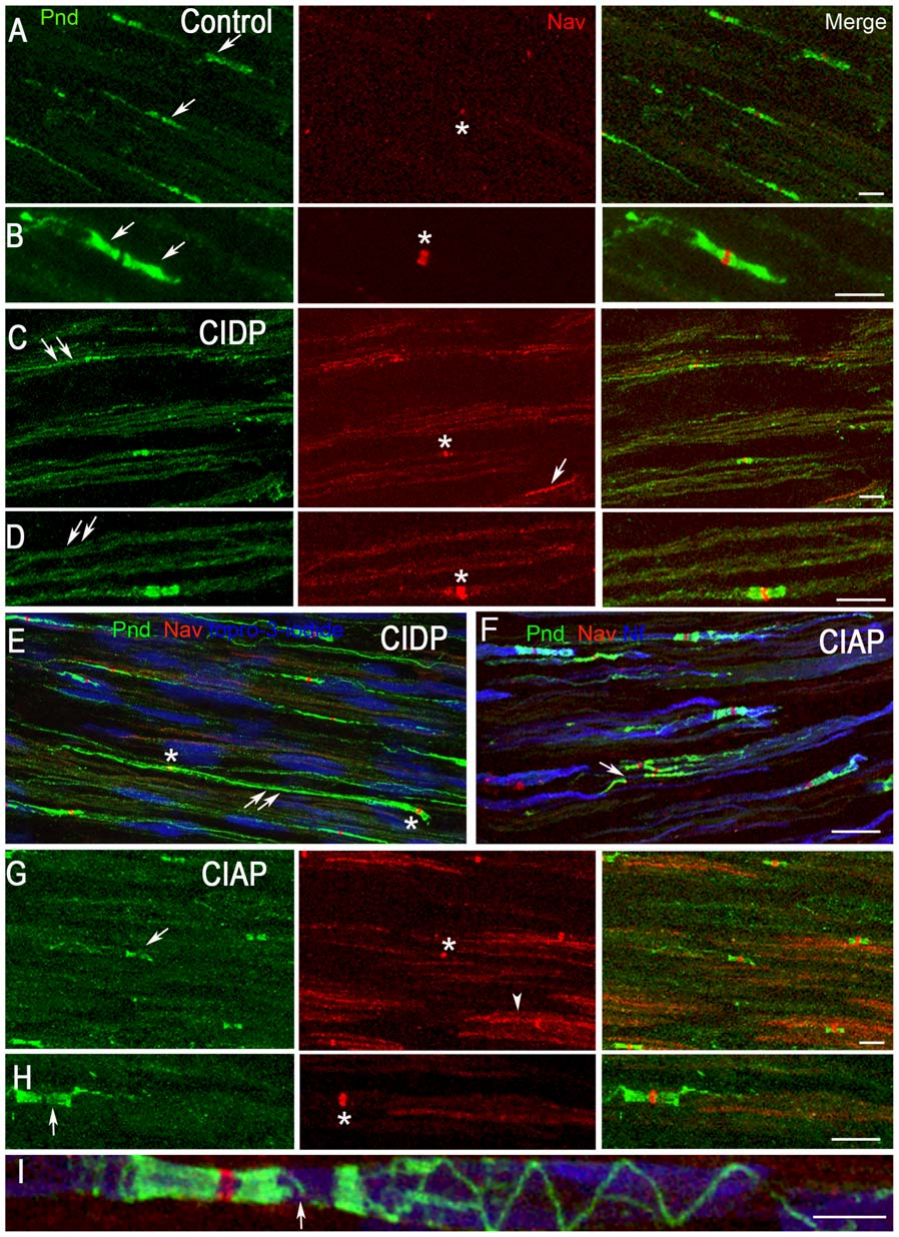
Alterations of nodal and paranodal proteins in chronic acquired neuropathies. Immunolocalization of paranodin (Pnd, green), voltage-gated Na^+^ channels (Nav, red) in longitudinal sections of superficial peroneal nerves (A–I). In blue is shown nuclear staining with topro-8 (E, I) and neurofilaments (Nf, F). B, D, and H are higher magnifications of A, C, and G, respectively. **A**, **B**. In a control nerve paranodin and Nav are correctly localized in the paranodal (arrows) and nodal (asterisks) regions, respectively. **C**, **D**. In CIDP (Patient 1), paranodin immunoreactivity extends to the internodal regions (double arrows). Nav immunoreactivity remains concentrated in some fibers (asterisk), while it has a diffuse, punctuate pattern along other fibers (arrow). **E**. Another CIDP case (Patient 6) with paranodin immunostaining (double arrows) extending in the internode. Nodal regions are indicated (asterisks). **F**. In CIAP (Patient 14), in some fibers the paranodal staining appears irregular (arrow). Neurofilaments are in blue. **G**, **H**. In CIAP patient 20, paranodin immunoreactivity is irregular in some nerve fibers with an asymmetrical localization (arrow), whereas the nodal expression of Nav appears normal (asterisk). A diffuse Nav-like immunoreactivity can be detected along some axons (arrowhead). **I**. In another CIAP Patient 13, an interrupted paranodal region (arrow) is visible at high magnification, followed by the normal paranodin staining along the mesaxon. Scale bar: A, C, E, F, G, 40 µm; B, D, H, 20 µm; I, 1.7 µm.

**Figure 5:**
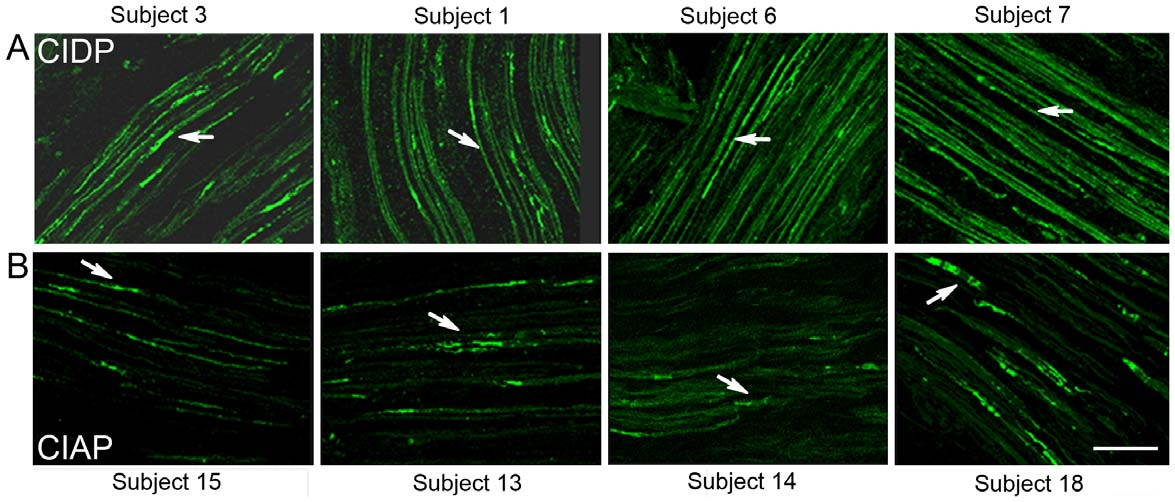
Comparison of paranodin immunoreactivity in several patients with CIDP and CIAP. Longitudinal sections from superficial peroneal nerves were immunostained with antibodies for paranodin in four patients with CIDP (A) and four patients with CIAP (B). The patient numbers are indicated on the figure. In CIDP samples paranodin immunoreactivity was visible along the internode in most fibers (A, arrows). In contrast in CIAP paranodin was restricted to the nodes (B, arrows), which appeared often asymmetrical or irregular. Scale bar 20 µm.

Since the nodal and paranodal regions were altered in CIDP and CIAP we examined the distribution of Nav that are located at nodes of Ranvier [22], and of paranodin, an axonal membrane glycoprotein highly enriched at paranodes [17] (Fig. 4). In the control nerve, Nav immunoreactivity was restricted to nodes of Ranvier (Fig. 4A, B, red) and flanked by paranodin immunoreactivity, which had a thin extension along the internal mesaxon (Fig. 4A, B, green), as reported in rodents [17], [23]. In CIDP, many fibers did not display the typical nodal/paranodal immunostaining described above since paranodin and Nav immunoreactivities had a punctuate appearance all along the axon (Fig. 4C, D), sometimes extending between two consecutive nodes (Fig. 4E). This diffuse distribution was apparent in samples from other patients (Fig. 5). In contrast, nodal regions appeared usually normal, some with particularly intense Nav immunofluorescence (Fig. 4C, D asterisk).

In CIAP nerves, paranodin immunoreactivity was distributed irregularly at paranodes, with areas of weak or absent staining (Fig. 4F–I). Nav enrichment at nodes appeared normal, although a diffuse, internodal, punctiform Nav-like immunoreactivity of variable intensity was also detected in the 10 patients examined (Fig. 4G, H, arrowhead). Paranodes were sometimes interrupted (Fig. 4G, H, I, arrow) and heminodes (Fig. 4F, arrow) or asymmetrical nodes were observed, more frequently in CIAP than in CIDP (18±1% of 230 nodes and 7.5±0.5% of 600 nodes, respectively). Other CIAP samples displayed a similar pattern of paranodin immunoreactivity, clearly different from that observed in CIDP (Fig. 5).

### KCNQ2 channels are altered in CIDP and CIAP

**Figure 6:**
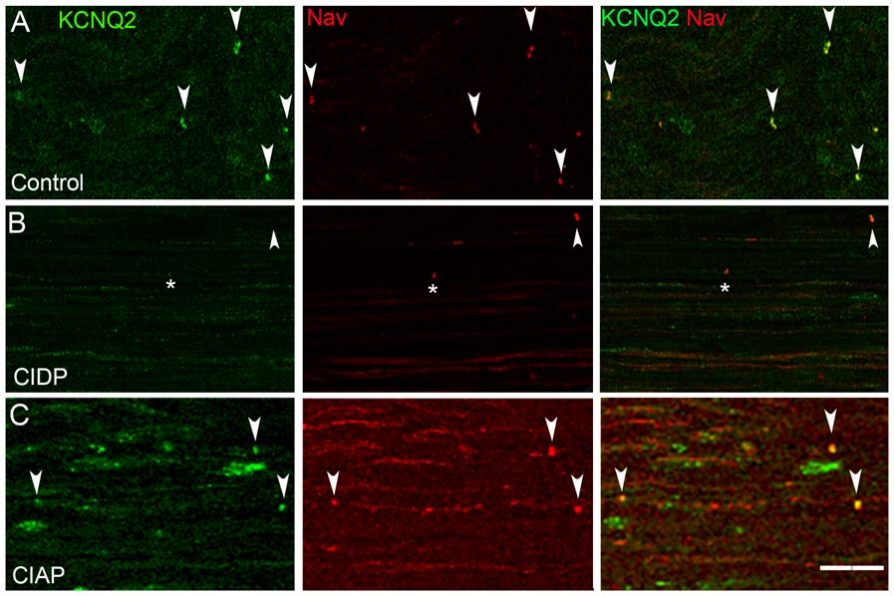
KCNQ2 immunoreactivity in a control nerve and in patients with CIDP and CIAP. Immunolocalization of KCQN2 (green) and voltage-gated Na^+^ channels (Nav, red) in longitudinal sections of superficial peroneal nerves. **A**. KCNQ2 and Nav immunoreactivities colocalize at the nodal region in the control nerve (arrowheads) and are not detected in the internodes. **B**. In a CIDP nerve (Patient 5), nodal KCNQ2 immunoreactivity is low (asterisk) or absent (arrowhead) and only a few nodes are labeled. A weak immunoreactivity is observed for the two ion channels in some internodes. **C**. In a CIAP sample (Patient 18), KCNQ2 and Nav colocalization is observed at nodes (arrowheads) but some immunoreactivity is also detected along the fibers. Scale bar: 20 µm.

We then examined the distribution of KCNQ2, a potassium channel subunit present in nodal regions [15]. As expected, KCNQ2 and Nav immunofluorescence were colocalized in nodal regions in control nerves (Fig. 6A). In the six CIDP nerves examined, the staining intensity of KCNQ2 was diminished or absent from nodes identified by Nav staining (Fig. 6B). In fibers displaying a diffuse punctuate distribution of Nav immunoreactivity, KCNQ2 immunoreactivity was also observed along the fibers (Fig. 6B). In eight CIAP nerves, KCNQ2 immunoreactivity colocalized with the nodal accumulations of Nav as in controls, but was also detected in internodal regions in some nerve fibers (Fig. 6C). Interestingly, wheras Nav and KCNQ2 immunoreactivities colocalized at nodes, it was not the case in the internodes.

### A mouse model of induced demyelination leads to alterations similar to those observed in CIDP

**Figure 7:**
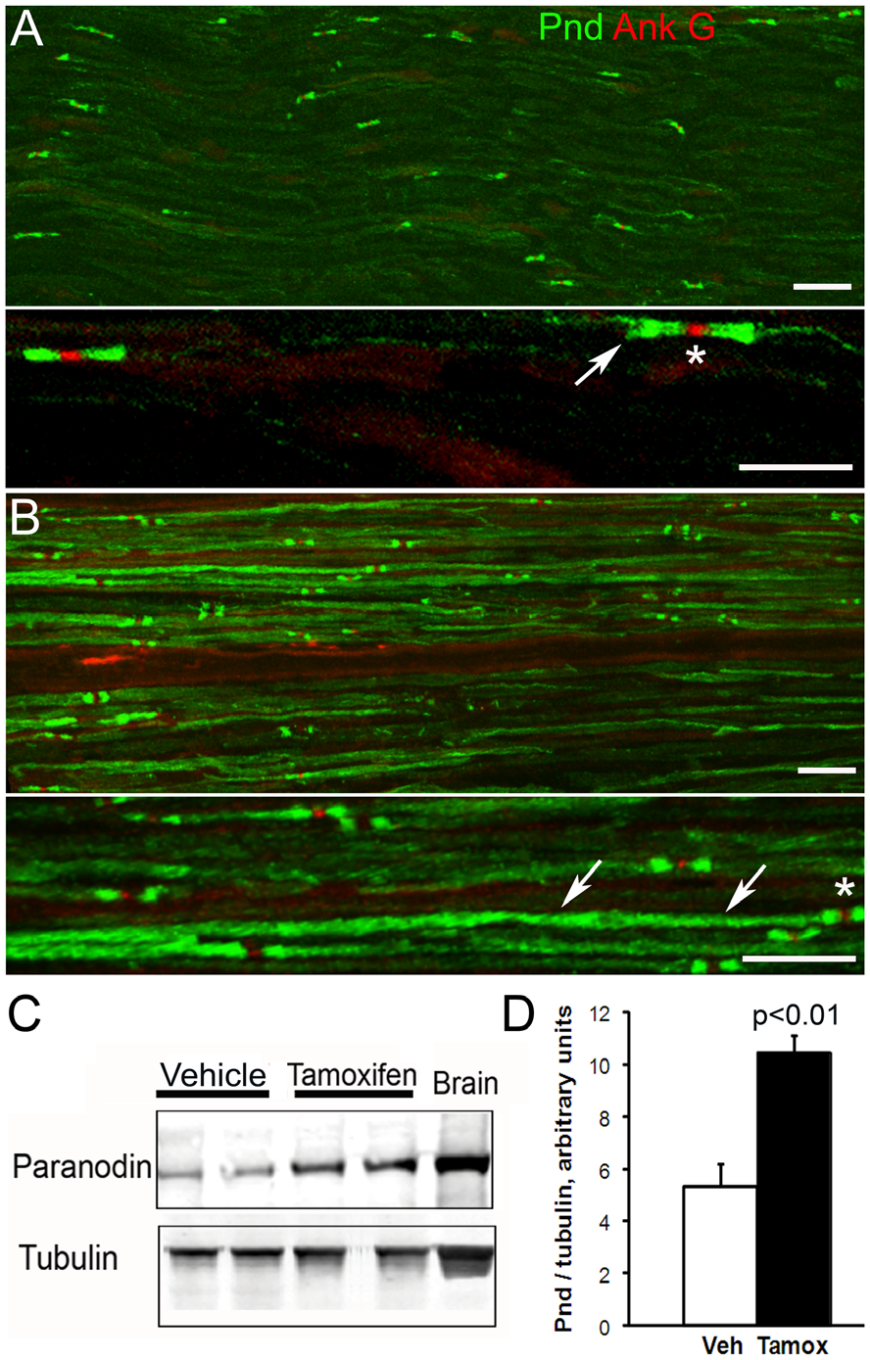
Distribution and levels of paranodin are increased in sciatic nerves in a mouse model of demyelination induced by conditional Krox 20 inactivation. Sciatic nerve sections were prepared 28 d after a 5-day treatment with vehicle (A) or tamoxifen (B) in *Krox20lacZ/flox*, *R26-CreER^T^* mice and immunolabeled for paranodin (Pnd, green) and Nav (red). **A**. In vehicle-injected animals, paranodin immunoreactivity is concentrated at paranodes (arrow) on both sides of the nodal Nav accumulations (asterisk). **B**. In tamoxifen-injected animals, paranodin immunoreactivity extends outside of the paranodal regions. In a fiber in which the position of the node is indicated by an asterisk, paranodin immunoreactivity is visualized all along the internodal region (arrowheads). In A and B two magnifications are shown. Scale bar: 45 µm for the upper panels and 20 µm for the lower panels. **C**. Western blot analysis of sciatic nerves extracts using an anti-paranodin antibody. Adult brain was analyzed for comparison. Alpha-tubulin was used for normalization of the quantity of protein. **D**. Quantification of immunoblot data obtained from sciatic nerves of 3 different mice (1 nerve per mouse) and in which paranodin immunoreactivity is normalized to alpha-tubulin. Statistical analysis with Student t-test.

In CIDP patients we observed an altered distribution of some neuronal proteins. Previous studies have shown that dysmyelination alters axonal domains organization, with the disappearance of paranodal accumulation of proteins [24], [25], [26]. In these studies no overall increase in paranodin immunoreactivity was described, in contrast to what was observed in patients with acquired CIDP. In order to better understand how the disappearance of differentiated Schwann cells alters axonal proteins in peripheral nerves, we used a mouse model in which demyelination is induced in the adult by a mechanism which targets specifically the Schwann cells [27]. This model is based on the tamoxifen-induced deletion of the *Krox20* gene, which codes for a transcription factor required for the maintenance of the expression of Schwann cell-specific genes. Induced demyelination in adult mice leads to the presence of macrophages degrading Schwann cells, onion bulbs, and abnormally thin myelin sheaths [27], which are reminiscent of alterations found in patients with CIDP. We examined paranodin and ankyrin G expression a month after tamoxifen treatment (Fig. 7). In vehicle-treated mice, paranodin and ankyrin G immunostaining were restricted to the paranodal and nodal regions, respectively (Fig. 7A). Paranodin immunofluorescence ended rather abruptly at the borders of the paranodal regions (Fig. 7A, arrow), with the exception of the labeling along the mesaxon. In tamoxifen-treated mice the limits of paranodin distribution were less regular and immunostaining extended into the internodal regions (Fig. 7B). Ankyrin G immunostaining was present in the nodal region but the density of these accumulations along the nerve fibers was higher in tamoxifen-treated (95 10^3^±10 10^3^ nodes per mm^3^ mean ± SEM, n = 3) than in vehicle-treated mice (38 10^3^±9 10^3^ nodes per mm^3^, n = 3, t-test p<0.05). To determine whether the increased immunofluorescence corresponded to increased levels of proteins, we used immunoblotting (Fig. 7C). The levels of paranodin were increased in sciatic nerve extracts from tamoxifen-injected mice, as compared to mice only injected with vehicle (Fig. 7C, D), showing that the induced demyelination increased the expression of paranodin.

**Figure 8:**
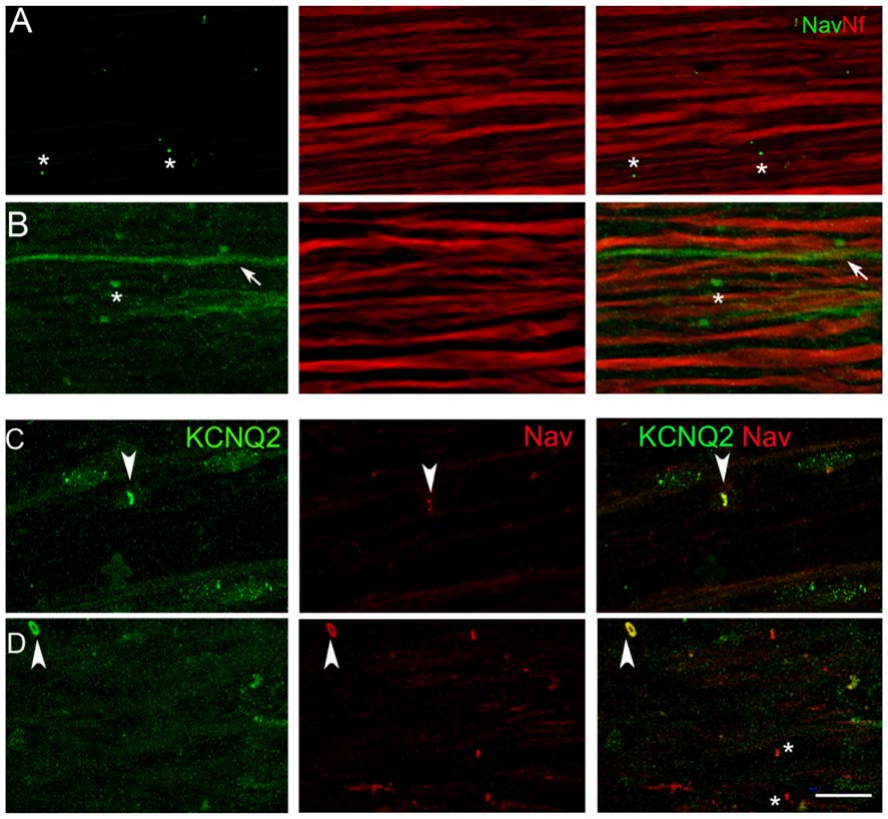
Distribution of voltage-gated Na^+^ channels (Nav) and KCNQ2 is altered in sciatic nerves of a mouse model of demyelination induced by conditional Krox 20 inactivation. Sciatic nerve sections were prepared 28 d after vehicle (A, C) or tamoxifen (B, D) injections in Krox20lacZ/flox, R26-CreERT mice. **A**. **B**. Immunolabeled for Nav (green) and phosphoneurofilament (Nf, antibody SMI31, red). In vehicle-injected animals, Nav expression is restricted to nodes (asterisks) (A). In tamoxifen-injected animals, Nav immunostaining is visible in nodal regions (asterisk) and, in some fibers, all along the axon (arrow) with a punctiform pattern (B). **C**, **D**. Longitudinal sciatic nerve sections were also doubly stained with antibodies for KCNQ2 (green) and Nav (red). In control vehicle-injected animals (C), KCNQ2 and Nav immunoreactivities colocalized at nodes of Ranvier (arrowhead). D. In tamoxifen-injected animals, KCNQ2 immunoreactivity is low and only a few nodes are co-stained (arrowhead) while in others KCNQ2 immunostaining is weak or absent (asterisks). Scale bar: 20 µm.

The distribution of Nav sodium channels was examined in double labeling experiments using a polyclonal anti-pan Nav antibody and the mouse monoclonal antibody SMI 31, which reacts with a phosphorylated epitope in neurofilaments H and, to a lesser extent, M. In vehicle-injected mice Nav immunoreactivity was concentrated in nodal regions identified as the narrowed region of the nerve fiber stained with SMI31 (Fig. 8A). In tamoxifen-treated mice Nav diffuse punctuate immunostaining extended all along some axons in which phosphoneurofilament labeling was very faint (Fig. 8B, arrow). We also compared the distribution of KCNQ2 and Nav in this mouse model. As expected, in vehicle-injected mouse KCNQ2 and Nav immunofluorescence were colocalized at nodes (Fig. 8C). In the tamoxifen-treated mice the staining intensity of KCNQ2 was diminished or absent in some nodeş while others were normal (Fig. 8D, arrowhead). A faint punctuate background immunoreactivity was also observed along fibers, which may indicate a diffuse distribution of KCNQ2.

### Paranodin immunofluorescence is a good marker for diagnosis of CIDP and CIAP

Since paranodin immunostaining appeared to be consistently different between the two types of chronic neuropathies (see Figs. 4 and 5), we tested whether it could be used as a tool for their differential diagnosis. For this purpose we used the 17 samples in which the sections had not been already used for the above studies. Randomly mixed images from 9 CIDP and 8 CIAP nerve sections stained with paranodin antibodies were submitted to four observers unaware of the diagnosis, used to examine immunofluorescence images but untrained for this specific task. They were requested to utilize the following criteria: 1/ For CIDP: high global intensity of paranodin immunostaining, and its extension beyond the paranodes (mesaxon excluded); 2/ For CIAP: low global intensity and/or interruption and/or presence of hemi-nodes. Only longitudinally well-oriented fibers were analyzed leading to the exclusion of one case which was considered to be non-interpretable. The percentage of correct diagnosis using these two criteria for the 16 interpretable samples ranged from 63% to 82% (mean ± SEM: 70±4). The percent agreement between at least three independent observers was 70%. These results show that examination of paranodin immunoreactivity on a single section provides a good indication for the diagnosis of CIAP vs CIDP.

## Discussion

The diagnosis of CIDP can be difficult and requires a combination of criteria, which includes in atypical cases histopathological features [5], [8], [10], in order to differentiate it from CIAP. In CIDP, demyelinating features include macrophage-mediated demyelination, hypomyelinated fibers, and onion bulbs [28], [29]. These lesions are variably observed and depend when the biopsy is performed in the course of the disease. CIAPs are characterized by variable axonal loss, features of axonal regeneration (regeneration clusters) and no or very few demyelinating lesions [30]. However, the difference between CIDP and CIAP may be difficult to establish [30], [31]. In the present study we investigated the existence of alterations of nodes of Ranvier in biopsies from patients with chronic acquired neuropathies since these regions are critical for the function of myelinated fibers and the site of early myelin changes in demyelination [32]. We then examined whether some of these alterations might help for the diagnosis.

Although electron microscopy of transverse sections of peripheral nerves is a classical approach that can contribute to the diagnosis of peripheral neuropathies, longitudinal sections are rarely, if ever, used. Nodes of Ranvier that are not easily investigated in transverse sections have not been characterized in these conditions. Here we specifically investigated the ultrastructure of nodes of Ranvier on longitudinal sections. Electron microscopy demonstrated the existence of alterations of nodal and paranodal regions in both CIDP and CIAP. As expected, alterations predominated in Schwann cells in CIDP, and in axons in CIAP. We also observed internodes with thin myelin or irregular myelination in CIAP, including heminodes, which were confirmed by counting after immunostaining. This finding is likely to correspond to regenerating fibers on which remyelination was taking place.

Immunofluorescence studies of nodal and paranodal proteins showed important alterations that were even more pronounced that what would have been expected from the electron microscopy study. These results suggest that organization of axonal domains is very sensitive to pathological alterations of both axons and Schwann cells. In CIAP, paranodin labeling of paranodes was irregular and interrupted, in relation to the ultrastructural findings showing axonal alterations. The relative nodal concentration of KCNQ2 and Nav channels was preserved, although some Nav immunoreactivity was detected all along the axons. This pattern of Nav channel immunostaining may correspond to the extensive zones of sodium channel staining reported along regenerating axons with ongoing remyelination after crushing the rat peroneal nerve [33]. Similar diffusion of Nav immunostaining has also been observed following inflammatory and partial axotomy lesion of the rat infraorbital nerve [34]. Internodal staining for Nav and KCNQ2 observed in our samples could also correspond in part to intra-axonal vesicles or vacuoles as detected by electron microscopy, although this could not be resolved by the methods used. Interestingly the two immunoractivities did not colocalize in the internodes, suggesting a different trafficking for these two channels. Thus, in CIAP the axonal pathology appears to combine abnormalities in nodal and paranodal regions and consequences of regeneration.

In CIDP, by contrast, paranodin immunofluorescence was increased and much more diffuse than in normal nerves. These modifications are likely to result directly from the Schwann cells alterations revealed by electron microscopy, since the correct localization of paranodin to the paranodal regions depends on myelinating glial cells [25], [26]. In central myelinated axons from patients with multiple sclerosis, demyelination results in a diffuse expression of paranodin [35] and its levels appear downregulated [36]. Delocalization of paranodin was also observed in Charcot-Marie-Tooth skin biopsies [21] and in dysmyelinating mutant mice [20], [25]. However, in contrast to these various conditions in which paranodin levels appeared normal or decreased, we found an increased paranodin immunoreactivity in CIDP, in addition to its delocalization. Nav expression appeared also increased in CIDP nerves and diffusely distributed in internodal regions of some axons. This is likely to result from the loss of axo-glial contact in demyelinated axons [37].

To further investigate the alterations of nodal and paranodal proteins induced by acquired demyelination, we studied mice in which Schwann cells dedifferentiation can be induced in adult animals [27]. These mice provide a unique model in which the genetically targeted and pharmacologically induced disappearance of a single transcription factor results in the disappearance of compact myelin. In contrast with toxic chemicals used for inducing demyelination there is neither Schwann cell death nor lesions of the axons. This model allows a specific investigation of the consequences of Schwann cells dedifferentiation on axonal proteins. As in CIDP, we observed an increased and diffuse paranodin immunolabeling with a diffusion of Nav immunoreactivity in some fibers. Increased levels of paranodin protein were demonstrated by immunoblotting, in contrast to what has been observed in dysmyelinating mutants [20], [25]. It is not known whether this corresponds to an increased production and/or a diminished destruction of the protein. Interestingly, we observed an increased density of ankyrin G clusters, presumably corresponding to nodes of Ranvier. This observation is likely to be related to the proliferation of Schwann cells reported in mice with tamoxifen-induced Krox20 deletion [27]. This would result in shorter internodes with an increased density of nodes or protonodes. In fact some nodes of Ranvier visualized by ankyrin G or Nav immunoreactivity appeared well preserved, in agreement with their slower alterations as compared to paranodes following dysmyelination [20], [25], [26]. In contrast, other nodes displayed a decreased KCNQ2 and Nav labeling, which may correspond to newly formed nodes attesting to the ongoing remyelination.

Since paranodin immunofluorescence is a robust marker of paranodes, differently altered in CIDP and CIAP, we tested whether it could be used as a help to the sometimes difficult diagnosis of these neuropathies. Using very simple criteria based on the intensity and distribution of paranodin immunoreactivity, we found that unaware observers were able to classify correctly biopsies as CIDP or CIAP in about 70% of the cases. It is important to note that these observers were not trained, and the inter-observer variability suggests that the training of the observers may improve the accuracy. Future studies will examine whether they can be applied to less invasive biopsy procedures such as muscle or skin biopsies. In summary, our study characterizes for the first time the profound alterations of nodes of Ranvier in human chronic acquired neuropathies using both electron microscopy and immunofluorescence, and shows that alterations in paranodin immunofluorescence may provide an interesting contribution to the diagnosis.

## Materials and Methods

### Patients

The present study was carried out using remaining material of biopsies previously carried out for diagnostic purposes. Patients diagnosed with CIDP and CIAP are described in Tables S1 and S2. Biopsies were carried out solely for diagnosis purposes before the present study was initiated and thus, did not need the approval from the ethics committee. Patients gave their written consent at the time of biopsy, for further use of possible remaining material for research purposes. Control nerves were a superficial peroneal nerve and terminal motor branches of intramuscular nerves in muscle biopsies devoid of lesion. Biological investigation included complete blood cell count, sedimentation rate, fasting glucose level, creatininemia, thyroid function, liver function, vitamin B12, folic acid, assay for anti-nuclear antibodies, complement, serologies for human immunodeficiency virus, B and C hepatitis, Lyme disease, search for cryoglobulinemia, monoclonal gammapathy, and cancer. Diagnosis of CIAP was made in patients with: i) Purely distal polyneuropathy, involving sensory (patients 19 and 21) or motor and sensory modalities (patients 13–18, 20, 22); ii) Electrophysiological findings of axonal degeneration; iii) Absence of abnormalities on biological investigation; iv) Slowly progressive course after a two-year follow up (patients 15 and 17 had a nerve biopsy less than two years after the beginning of the disease but further follow-up did not change the diagnosis); v) Absence of the Ad Hoc Subcommittee of the American Academy of Neurology (AAN) criteria for nerve demyelination and absence of other identifiable cause of polyneuropathy (absence of vasculitis, granuloma or abnormal deposits) [28]. Diagnosis of CIDP was based on typical clinical and ENMG findings in patient 1 only, and in the others on: i) Very suggestive clinical presentation with proximal limbs involvement (patients 5, 6), pronounced ataxia (patients 3, 4, 5), diffuse areflexia (patients 5, 8, 11), relapsing course (patient 6), involvement of cranial nerves (patients 7, 8) and ii) presence of some ENMG criteria for demyelination (patients 2, 7, 9). These suggestive combination of clinical and electrophysiological criteria led to measure the CSF protein content, which was abnormally high in patients 2, 4, 6, 7, 9, and 12, and to perform a nerve biopsy, which disclosed the AAN Ad Hoc Subcommittee histological criteria for demyelination [28] in patients 2, 4, 6, 8, and 10–12. Fifty eight percent of the patients (patients 1–3, 5, 7, 8, and 10) responded favorably to immunomodulating treatment.

### Mice

Mice with conditional Krox20 inactivation were produced as described [27]. In brief, these mice carried one allele in which Krox20 coding sequence was replaced by beta-galactosidase sequence (lacZ) and one allele in which LoxP sequences allowed the excision of a critical fragment of the Krox20 coding sequence following the action of Cre (floxed gene). These mice also carried a transgene, *R26-CreER^T^*, coding for Cre fused to a mutated form of the estrogen receptor (ER^T^) able to bind tamoxifen but not endogenous estrogens, which prevents the nuclear activity of Cre. For inducing *Krox20* inactivation, *Krox20^lacZ/flox^*, *R26-CreER^T^* mice were injected with tamoxifen (10 mg/ml, i.p.), in a 10∶1 sunflower oil/ethanol solution, 100 µl twice a day for 5 days. Control mice received only vehicle. Mice were killed and nerves prepared 28 days after this treatment. The total number of mice used was six and all experiments were in accordance with the guidelines of the French Agriculture and Forestry Ministry for handling animals (decree 87849, license B75-05-22). Our Institute has the authorization for the use of mice without approval of specific research project.

### Biopsy nerve preparation and electron microscopy

Superficial peroneal nerves were obtained from patients for diagnosis purposes, previously to the present study, under local anesthesia, at the lower third lateral part of the leg. One centimeter was fixed in 2.5% (weight/vol) glutaraldehyde in sodium phosphate buffer (pH 7.4, 150 mM), post-fixed in 2% (weight/vol) osmium tetroxide in the same buffer and embedded in Epon. Semi-thin sections (0.5 µm) were stained with toluidine blue and examined under light microscope. One centimeter was orientated longitudinally, mounted on a cork disk with tragacanth gum and frozen in isopentane cooled by liquid nitrogen at −160°C. Electron microscopy was carried out on 2 samples from patients with CIAP and 2 with CIDP at the *Institut du Fer à Moulin* Cell and Tissue Imaging Facility with a Philips CM 100 microscope equipped with a Gatan Orius 832 camera for digitized acquisition. Nodal regions were located at low magnification and extensively studied at higher magnification.

### Antibodies

Rabbit antibodies against paranodin (L51) have been described [17]. Voltage-gated Na^+^ channel α subunit mouse monoclonal antibody (PAN Nav, clone K58/35) and rabbit polyclonal anti-sodium channel (PAN-SP19) were from Sigma-Aldrich (St Louis, MO). Rabbit antibodies for KCQN2 [15] were a gift of Jérôme Devaux (CNRS, Marseille, France). Rabbit antibodies for ankyrin G [38] and neuronal class III β-tubulin (TUJ1), and mouse monoclonal antibody for ankyrin G were from Chemicon Inc. (Temecula, CA).

### Tissues extracts and immunoblotting

Mouse sciatic nerves were dissected out and frozen in isopentane pre-cooled with liquid nitrogen. Samples were stored at −80°C. Tissue fragments (∼1 mm^3^) from frozen nerves were homogenized in a buffer containing 10 mM sodium phosphate, pH 7.8, 60 mM NaCl, 1% (vol/vol) Triton X-100, 0.5% (weight/vol) deoxycholic acid, 0.1% (weight/vol) SDS, 10% (vol/vol) glycerol, 25 mM alpha-glycerol-phosphate, 50 mM sodium fluoride, 2 mM sodium pyrophosphate, 1 mM sodium orthovanadate, and Complete protease inhibitor (Roche Applied Science). Insoluble material was removed by centrifugation at 21,000×g for 10 min at 4°C. Protein concentration was determined using microBCA (Pierce). Extracts were in NuPAGE LDS were resolved on 4–12% NuPAGE BisTris gels (Invitrogen), and transferred to nitrocellulose membranes. Membranes were blocked and incubated with antibodies as described [39]. Primary antibodies were detected using IRDye 700- or 800-conjugated secondary antibodies (Rockland) and Odyssey Imaging System (LI-COR biosciences).

### Immunofluorescence

Longitudinal sections of frozen human superficial peroneal nerve biopsies or mouse sciatic nerves were cryostat-sectioned (8–10 µm), fixed in methanol-acetone (1∶1), washed in phosphate-buffered saline (PBS), blocked in PBS with 30 g/L bovine serum albumin), and incubated overnight at 4°C with the primary antibody in blocking buffer. After washing in PBS with 0.01% (vol/vol) Tween 20, sections were incubated 1 hour at room temperature with Alexa fluor 488 donkey anti-rabbit IgG (Molecular Probes), Cy3 goat anti-mouse IgG (H+L) highly cross-adsorbed, or Cy5 goat anti-chicken IgG (Jackson Laboratories) diluted 1/500 in blocking solution. Sections were washed in distilled water and mounted in Vectashield.

Immunofluorescence analysis was carried out at the *Institut du Fer à Moulin* Cell and Tissue Imaging facility, using a Leica epifluorescence microscope equipped with a CCD camera (Micromax Roper Scientific), or a Leica SP2 confocal laser scanning microscope (Leica, Mannheim, Germany). Acquisition features were kept constant for all acquisitions with the same antibodies.

### Validation of paranodin immunofluorescence as a diagnostic marker

Images from biopsies of CIAP (8) or CIDP (9) immunostained with paranodin antibodies were coded, randomly mixed and examined by 4 unaware observers who were asked to classify them into 3 categories (probable CIAP, probable CIDP, unknown) according to the intensity and extension of paranodin immunostaining. The proportion of correct identification was determined for each disease.

## Supporting Information

Figure S1 Ultrastructure of a node of Ranvier in superficial peroneal nerve from a CIAP patient. A. Low magnification of a longitudinal ultrathin section showing the nodal and paranodal regions. B. Higher magnification of A showing normal perinodal microvilli emanating from the Schwann cells (arrow). C. Higher magnification of A showing paranodal loops with septate-like axoglial junctions (arrowhead). Scale bars: A: 1 µm, B: 0.7 µm and C: 0.5 µm.
(3.82 MB TIF)

Table S1 Clinical, paraclinical, and histological characteristics of CIDP patients.
(0.02 MB DOCX)

Table S2 Clinical and histological characteristics of patients with CIAP.
(0.02 MB DOCX)
