# Supplementary figures and images for: Nodes of Ranvier and Paranodes in Chronic Acquired Neuropathies
Source: PLoS One. 2011 Jan 18;6(1):e14533. doi: 10.1371/journal.pone.0014533 (PMC3022580; doi:10.1371/journal.pone.0014533)

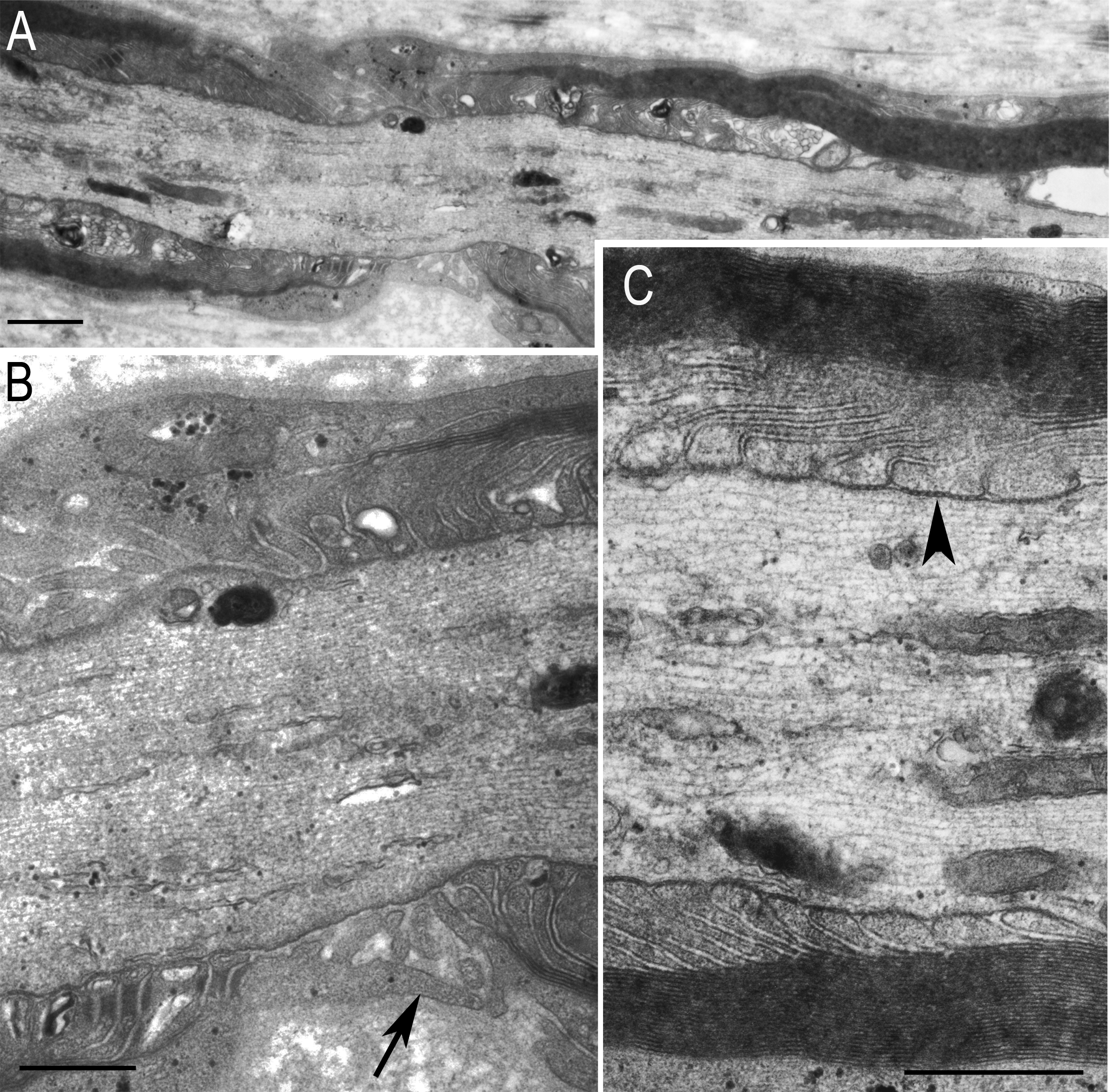

Supplement: Figure S1 — Ultrastructure of a node of Ranvier in superficial peroneal nerve from a CIAP patient. A. Low magnification of a longitudinal ultrathin section showing the nodal and paranodal regions. B. Higher magnification of A showing normal perinodal microvilli emanating from the Schwann cells (arrow). C. Higher magnification of A showing paranodal loops with septate-like axoglial junctions (arrowhead). Scale bars: A: 1 µm, B: 0.7 µm and C: 0.5 µm. (3.82 MB TIF) [file pone.0014533.s001.tif]
