# Supplementary material for: Nodes of Ranvier and Paranodes in Chronic Acquired Neuropathies
Source: PLoS One. 2011 Jan 18;6(1):e14533. doi: 10.1371/journal.pone.0014533 (PMC3022580; doi:10.1371/journal.pone.0014533)
Supplement: Table S1 — Clinical, paraclinical, and histological characteristics of CIDP patients. (0.02 MB DOCX) [file pone.0014533.s002.docx]

**Supplementary Table 1: Clinical, paraclinical, and histological characteristics of CIDP patients**

| **Patient #** | **Gender** | **Age at biopsy (y)** | **Disease duration** | **Symptoms at onset** | **Clinical data** | | | | | | | | | | | **CSF**  **protein (g/l)** | **Histological data** | | | | |
| --- | --- | --- | --- | --- | --- | --- | --- | --- | --- | --- | --- | --- | --- | --- | --- | --- | --- | --- | --- | --- | --- |
|  |  |  |  |  | **Distal motor deficit** | | **Proximal motor deficit** | | **Superficial sensory loss** | | **Deep sensory loss** | | **Osteo-tendinous reflexes** | | **Response to treatment** |  | **Myelinated**  **fiber loss** | **Regenerative clusters** | **Inflam-**  **matory infiltrates** | **Hypo-myelinated fibers** | **Onion bulbs** |
|  |  |  |  |  | **UL** | **LL** | **UL** | **LL** | **UL** | **LL** | **UL** | **LL** | **UL** | **LL** |  |  |  |  |  |  |  |
| 1 | M | 44 | 10 mo | Numbness in the feet, left LL weakness | + | – | + | + | + | + | + | + | – | – | + | 6 | + | UK | + | UK | UK |
| 2 | M | 57 | 5 y | Distal LL paresthesias | – | – | – | – | – | + | – | + | + | + | + | 0.97 | +++ | + | + | + | ++ |
| 3 | F | 73 | 3 y | Steppage  Distal LL paresthesias and burning Imbalance | – | + | – | – | – | + | – | + | + | – | + | <0.1 | + | + | + | + | – |
| 4 | M | 63 | 5 y | Difficulties for walking  Paresthesias in the feet and in the right hand  Imbalance | – | + | – | – | – | + | – | + | + | –  (Ach) | – | 0.64 | ++ | ++ | + | ++ | + |
| 5 | F | 51 | 2 y | Difficulties for walking, paresthesias in LL then in UL, imbalance | + | + | + | + | + | + | + | + | **–** | **–** | + | 0.49 | + | + | – | + | – |
| 6 | M | 69 | 2 mo | Paresthesias and burning in LL,  LL weakness | **–** | + | **–** | + | **–** | + | **–** | **–** | + | + | UK | 0.66 | ++ | + | + | **+** | + |
| 7 | M | 28 | 3 mo | Numbness in the face and in the tongue, burning of the feet and in the right hand | **–** | **–** | **–** | **–** | **+** | + | **–** | **–** | + | + | + | 1.38 | +++ | + | + | + | **–** |
| 8 | M | 53 | 5 y | Paresthesias in the hands and in the face | **–** | **–** | **–** | **–** | + | + | **–** | **+** | **–** | **–** | + | N | + | ++ | + | + | + |
| 9 | M | 64 | 5 y | Numbness in the right and then the left foot | **–** | **–** | **–** | **–** | **–** | **+** | **–** | **+** | + | –  (Ach) | – | > 1 | ++ | UK | **–** | UK | – |
| 10 | M | 51 | 5 y | Numbness in the right and then the right foot | **_** | **_** | **_** | **_** | **+** | **+** | **+** | **+** | + | _  (Ach) | + | UK | ++ | +++ | **_** | ++ | _ |
| 11 | M | 77 | 2 y | Distal paresthesias of the four limb | **_** | **_** | **_** | **_** | **+** | **+** | **_** | **+** | _ | _ | _ | 0.48 | ++ | +++ | **_** | + | ++ |
| 12 | M | 79 | 10 y | Paresthesias and burning pain in the right foot and then in the left foot | **_** | **_** | **_** | **_** | **+** | **+** | **_** | **+** | + | _ | UK | 0.58 | ++ | + | **_** | + | ++ |

y: years; mo: months; N: normal; UK: unknown; UL: upper limbs; LL: lower limbs; +: presence; –: absence; Ach: Achilles tendon.

Myelinated fiber loss: +: discrete; ++: moderate; +++: severe. Regenerative clusters, hypomyelinated fibers and onion bulbs: -: absence, +: <5, ++: 5 to 10, +++: > 10.

| **Patient #** | **Gender** | **Age at biopsy (y)** | **Disease duration** | **Symptoms**  **at onset** | **Clinical data** | | | | | | | | | | **Histological data** | | | | |
| --- | --- | --- | --- | --- | --- | --- | --- | --- | --- | --- | --- | --- | --- | --- | --- | --- | --- | --- | --- |
|  |  |  |  |  | **Distal motor deficit** | | **Proximal motor deficit** | | **Superficial sensory loss** | | **Deep sensory loss** | | **Osteo-tendinous reflexes** | | **Myelinated fiber loss** | **Regenerative clusters** | **Inflammatory infiltrates** | **Hypomyelinated fibres** | **Onion bulbs** |
|  |  |  |  |  | **UL** | **LL** | **UL** | **LL** | **UL** | **LL** | **UL** | **LL** | **UL** | **LL** |  |  |  |  |  |
| 13 | M | 72 | 7 | Steppage  LL pain | – | + | – | – | – | + | – | + | + | – | +++ | +++ | – | – | **–** |
| 14 | F | 70 | 5 | Steppage  Imbalance | – | + | – | – | – | + | – | + | + | – | +++ | +++ | – | – | **–** |
| 15 | F | 67 | 1 | Fatigability for walking  Cramps | + | + | – | – | – | + | – | + | – | – | ++ | ++ | – | + | **–** |
| 16 | F | 70 | 2 | Steppage  Paresthesias of the feet | – | + | – | – | – | + | – | + | + | – | ++ | ++ | – | – | **–** |
| 17 | M | 75 | 0.8 | Steppage  Paresthesias of the toes | – | + | – | – | – | + | – | – | + | – (Ach) | +++ | ++ | – | – | **–** |
| 18 | M | 91 | 5 | Imbalance | – | + | – | – | – | + | – | + | + | – (Ach) | +++ | ++ | + | – | **–** |
| 19 | M | 47 | 10 | Plantar dysesthesias,  LL pain | – | – | – | – | + | + | – | + | + | – (Ach) | ++ | +++ | + | – | **–** |
| 20 | M | 79 | 2 | Paresthesias, burning  LL pain | – | + | – | – | – | + | – | – | + | – | ++ | ++ | – | – | **–** |
| 21 | F | 80 | 5 | Distal paresthesias, LL pain, imbalance | _ | _ | _ | _ | _ | + | _ | + | _ | _ | ++ | ++ | _ | + | **_** |
| 22 | M | 71 | 3 | Steppage,  LL pain | _ | + | _ | _ | _ | + | _ | + | + | _ | ++ | ++ | _ | _ | **_** |

**Supplementary Table 2: Clinical and histological characteristics of patients with CIAP**

UL: upper limbs; LL: lower limbs; +: presence; –: absence; Ach: Achilles tendon. Myelinated fibre loss: +: discrete; ++: moderate; +++: severe. Regenerative clusters and hypomyelinated fibers: -: absence, +: <5, ++: 5 to 10, +++: > 10.
