# Supplementary material for: Nodes of Ranvier and Paranodes in Chronic Acquired Neuropathies
Source: PLoS One. 2011 Jan 18;6(1):e14533. doi: 10.1371/journal.pone.0014533 (PMC3022580; doi:10.1371/journal.pone.0014533)
Supplement: Table S2 — Clinical and histological characteristics of patients with CIAP. (0.02 MB DOCX) [file pone.0014533.s003.docx]

| **Patient #** | **Gender** | **Age at biopsy (y)** | **Disease duration** | **Symptoms**  **at onset** | **Clinical data** | | | | | | | | | | **Histological data** | | | | |
| --- | --- | --- | --- | --- | --- | --- | --- | --- | --- | --- | --- | --- | --- | --- | --- | --- | --- | --- | --- |
|  |  |  |  |  | **Distal motor deficit** | | **Proximal motor deficit** | | **Superficial sensory loss** | | **Deep sensory loss** | | **Osteo-tendinous reflexes** | | **Myelinated fiber loss** | **Regenerative clusters** | **Inflammatory infiltrates** | **Hypomyelinated fibres** | **Onion bulbs** |
|  |  |  |  |  | **UL** | **LL** | **UL** | **LL** | **UL** | **LL** | **UL** | **LL** | **UL** | **LL** |  |  |  |  |  |
| 13 | M | 72 | 7 | Steppage  LL pain | – | + | – | – | – | + | – | + | + | – | +++ | +++ | – | – | **–** |
| 14 | F | 70 | 5 | Steppage  Imbalance | – | + | – | – | – | + | – | + | + | – | +++ | +++ | – | – | **–** |
| 15 | F | 67 | 1 | Fatigability for walking  Cramps | + | + | – | – | – | + | – | + | – | – | ++ | ++ | – | + | **–** |
| 16 | F | 70 | 2 | Steppage  Paresthesias of the feet | – | + | – | – | – | + | – | + | + | – | ++ | ++ | – | – | **–** |
| 17 | M | 75 | 0.8 | Steppage  Paresthesias of the toes | – | + | – | – | – | + | – | – | + | – (Ach) | +++ | ++ | – | – | **–** |
| 18 | M | 91 | 5 | Imbalance | – | + | – | – | – | + | – | + | + | – (Ach) | +++ | ++ | + | – | **–** |
| 19 | M | 47 | 10 | Plantar dysesthesias,  LL pain | – | – | – | – | + | + | – | + | + | – (Ach) | ++ | +++ | + | – | **–** |
| 20 | M | 79 | 2 | Paresthesias, burning  LL pain | – | + | – | – | – | + | – | – | + | – | ++ | ++ | – | – | **–** |
| 21 | F | 80 | 5 | Distal paresthesias, LL pain, imbalance | _ | _ | _ | _ | _ | + | _ | + | _ | _ | ++ | ++ | _ | + | **_** |
| 22 | M | 71 | 3 | Steppage,  LL pain | _ | + | _ | _ | _ | + | _ | + | + | _ | ++ | ++ | _ | _ | **_** |

**Supplementary Table 2: Clinical and histological characteristics of patients with CIAP**

UL: upper limbs; LL: lower limbs; +: presence; –: absence; Ach: Achilles tendon. Myelinated fibre loss: +: discrete; ++: moderate; +++: severe. Regenerative clusters and hypomyelinated fibers: -: absence, +: <5, ++: 5 to 10, +++: > 10.
